# Evaluation of the Cytotoxic Effect of the Brittle Star *(Ophiocoma Erinaceus)* Dichloromethane Extract and Doxorubicin on EL4 Cell Line

**Published:** 2017

**Authors:** Mahbubeh Afzali, Javad Baharara, Khadijeh Nezhad Shahrokhabadi, Elaheh Amini

**Affiliations:** a *Department of Biology, Mashhad Branch, Islamic Azad University, Mashhad, Iran.*; b *Research Center for Animal Development Applied Biology & Biology Department, Mashhad Branch, Islamic Azad University, Mashhad, Iran. *; c *Department of Biology, Mashhad Branch, Islamic Azad University, Mashhad, Iran.*; d *Department of Cellular & Molecular Biology, Faculty of Biological Sciences, Kharazmi University, Tehran, Iran.*

**Keywords:** Apoptosis, Leukemia, Marine invertebrates, Cytotoxic effect

## Abstract

Leukemia is a blood disease that creates from inhibition of differentiation and increased proliferation rate. The nature has been known as a rich source of medically useful substances. High diversity of bioactive molecules, extracted from marine invertebrates, makes them as ideal candidates for cancer research. The study has been done to investigate cytotoxic effects of dichloromethane brittle star extract and doxorubicin on EL4 cancer cells. Blood cancer EL4 cells were cultured and treated at different concentrations of brittle star **(*Ophiocoma erinaceus*)** dichloromethane extract at 24, 48 and 72 h. Cell toxicity was studied using MTT assay. Cell morphology was examined using an invert microscope. Further, apoptosis was examined using Annexin V-FITC, propodium iodide, DAPI, and Acridine orange/propodium iodide staining. Eventually, the apoptosis pathways were analyzed using measurement of Caspase-3 and -9 activity. The statistical analysis was performed using SPSS, ANOVA software, and Tukey’s test. *P*<0.05 was considered to be significant. MTT assay and morphological observations showed that dichloromethane extract can inhibit cell growth in a dose dependent. The results considered 32 µg/mL of the extract as IC_50_. Also, doxorubicin suppressed EL4 proliferation as IC_50_=32 µg/mL. All experiments related to apoptosis analysis confirmed that dichloromethane brittle star extract and doxorubicin have a cytotoxic effect on EL4 cells inIC_50_ concentration. The study showed that dichloromethane brittle star extract is as an adjunct to doxorubicin in treatment of leukemia cells.

## Introduction

Cancer is a chronic disease and the second important cause of human mortality in the world (1). Leukemia, a type of blood cancer, rises by enhancing number of unusual white blood cells identified by suppression of differentiation and rate of proliferation induction (2, 3). Leukemia has been divided into four main categories, including chronic myelogenous leukemia (CML), acute myelogenous leukemia (AML), chronic lymphocytic leukemia (CLL), and acute lymphocytic leukemia (ALL) (4).

There are some treatments for leukemia including surgery, radiation therapy, immunotherapy and chemotherapy, although, none of these treatments are definite for the cancer (5). Therefore, searching for new efficient chemotherapeutic agents, as the treatment for leukemia, seems promising for some time (6). Doxorubicin, an anthracycline antibiotic, has been usually used as a treatment for several cancers such as solid tumors, breast cancer, lymphomas, and leukemia (7). Unfortunately, the application of Doxorubicin has been limited because of its side effects (8).

Natural metabolites are useful as the treatment for various human diseases (9). They are known potentially useful substances in pharmacology and can be used as treatment for some diseases such as cancer with minimal side effects; more than 50% of all present drugs have been made using natural products. Some of these substances can control cancer cell development with apoptosis induction (10).

Natural products have been important sources of new anti-cancer drugs (11). Marine biodiversity is the unique. Many secondary metabolites have been evolved in marine organisms making them successful in adaptation against environmental pressures, competition for space and niche, defending against predators and reproduction, which cannot be found in terrestrial habitats (12). Apoptosis and necrosis have been known the two mechanisms destroying cancer cells (13). Apoptosis cell deaths in contrast with necrosis do not recruit cell inflammatory responses that cause normal cell lysis. Therefore, it is crucial to increase apoptosis cell deaths during chemotherapeutic treatment of cancer to overcome necrosis complication (2).

Searching for novel drugs seems important in cancer treatment, because most cancers have been resisted against chemotherapeutic drugs (14). Further, the high toxicity has been generally observed in some cancer chemotherapeutic drugs that can make serious side effects. Therefore, request for novel anti-cancer drugs with lowest toxicity slightest side-effects and higher therapeutic efficiency is increasing. Recent studies have been demonstrated that natural products possess valuable biomedical properties (15). 

Marine invertebrates (Porifera, Cnidaria, Arthropoda, Mollusca, Echinodermata, etc.) are one of the main groups of marine organisms. Nowadays, various ranges of secondary metabolites have been extracted from marine invertebrates that are extremely useful to produce marine drugs (16).

Echinoderms have comprised a diverse phylum of marine invertebrates dividing into five classes, including Asteroidea (sea stars, or starfish), Crinoidea (crinoids), Ophiuroidea (brittle stars), Echinoidea (sea urchins), and Holothuroidea (sea cucumbers) (17). Sea stars have been famed more than other echinoderms for their secondary metabolites such as saponins, which have significant cytotoxic effects (18). *Ophiocoma erinaceus* is one of Persian Gulf brittle stars with colorful long body and tiny spines on the arms. There is a little information available on pharmaceutical effects of secondary metabolites of the species. So, due to the direct relationship of sea stars and brittle stars, this study has been chosen to evaluate the combination cytotoxic effect of doxorubicin and the Persian Gulf brittle star dichloromethane extract against leukemia cell line.

## Experimental

*Materials*

EL4 blood cancer cells were purchased from NCBI (National Cell Bank of Iran). RPMI-1640 and Trypan blue were purchased from Bio idea (Iran).Trypsin/EDTA (1X) and Fetal Bovine Serum were provided from GIBCO (USA).Penicillin/streptomycin and Phosphate Buffered Saline were purchased from PAA (Austria). Annexin V-FITC kit, Caspase-9 assay, and Caspase-3 assay kits were purchased from Abcam (UK), PI. DAPI kits, MTT [3-(4, 5-dimethylthiozol-2-il) 2,5dipheniltetrazoliumbromide], and Acridine orange/propodium iodide were purchased from SIGMA (USA). This experiment was performed at Research Center laboratory of Applied Biology at t Mashhad Branch of the Islamic Azad University in 2013.

*Preparation of* *brittle star* *dichloromethane* *extract*

Firstly, morphometric characters of *O. erinaceus* were examined. Identified specimens were washed and dried at room temperature in the darkness. Then, 10 mL of methanol per gram was added to the samples and placed on a rotating shaker for 3 days. Later, the extract was filtered using 11 µm Whatman filtering paper and concentrated using a vacuum evaporator. Then the concentrated fluid extracted by dichloromethane/water (3 to1), and after the desired period two parts consist of dichloromethane and water layer were separated, and the dichloromethane portion was placed in the vacuum to be concentrated. Finally, this extract was stored in –20°C until next experiments.

*Cell culture*

The EL4 Cell line was obtained from Pasteur Institute of Iran. Cells were cultured in RPMI 1640 medium supplemented with 12% Fetal Bovine Serum and 1% penicillin/streptomycin, and then incubated at 37°C in a humidified atmosphere containing 5% CO2.

*MTT assay*

El4 cells were seeded in 96-well plates and treated with different concentrations of brittle star dichloromethane extract*,* Doxorubicin and a combination of both of them for 24, 48 and 72 h. Then, MTT (5 mg mL^-^^1^ in phosphate buffered saline (PBS) was added to the wells and incubated at 37°C in the darkness for 4 h and DMSO was added to totally dissolve the formazan crystals. The absorbance of each well was measured at a wavelength of 560 nm with a Spectrophotometer. The cell viability inhibition was calculating using this equation: 

Cell viability (%) =Absorbance in test wells/Absorbance in control wells×100

*Annexin V-FITC*

The appearance of phosphatidyl serine (PS) on the extracellular side of membrane was evaluated using Annexin/PI method. Cells were suspended in 500 µL 1X Binding Buffer, after treating with different concentrations of brittle star dichloromethane extract and centrifuging, according to the company’s protocol. Then, 5 µL of Annexin V-FITC and 5µL of propodium iodide were added to centrifuged cells, and then they were placed for5 min at room temperature in darkness. Finally, they were analyzed on flow cytometer.

*PI staining*

The cells were treated with different concentrations of brittle star dichloromethane extract, and the combination of brittle star dichloromethane extract and Doxorubicin for 24 h and then were centrifuged. In the next step 700 µL PI solution was added to inside the wells and incubated at 37 °C for 20 min in the darkness. Then, analysis was done using flow cytometry.

*Acridine orange/propodium iodide staining*

The EL4 Cell line cells were treated with different concentrations of brittle star dichloromethane extract, Doxorubicin, and a combination of both of them for 24 h. The untreated cells and the treated cells were washed using PBS and stained using Acridine orange (20 μg/mL) and propodium iodide (20 μg/mL) (1:1). Finally, the cells were immediately mounted on slides and examined using a fluorescence microscope to determine morphology of the cells undergoing apoptosis.

*DAPI staining*

The cells were treated using different concentrations of brittle star dichloromethane extract and were incubated for 24 h. Then, the cells were washed twice using PBS. DAPI was added to the cells and incubation was done for 10 min in the darkness. Again, the cells were washed twice using PBS and then were suspended in 1000 μL of methanol. The morphology of nuclei was observed using a Fluorescence microscopy.

*Caspase-3 and Caspase-9 assays*

The assay was performed using a Caspase-9, or Caspase-3, apoptosis detection, colorimetric bioassay kit (Abcam, UK), according to the company’s protocol. EL4 cells (2–5x10^6^) were treated with 31, and 62 µg/mL of the extract for 24 h. The untreated and treated cells were suspended in 50 µL of cell lysis buffer (supplied with the kit) and incubated on ice for 10 min. After centrifuging at 10,000 x g for one min in 4°C, the supernatants were moved to new tubes and stored on ice. The Caspase-9 and Caspase-3 were done, according to the supplied kit protocol. Fifty µL of 2X reaction buffer (containing 10 mM DTT) and 5 µL of LEHD-pNA substrate (4 mM) (200 µM final concentration) was added to each sample and incubated at 37 °C for 1–2 h. Absorbance was read at 405 nm using a spectrophotometer and calculations were done.

*Data analysis*

The statistical analysis was performed using SPSS, one way ANOVA software, Tukey’s test. P<0.05 was considered to be significant.

## Results and Discussion

*MTT assay*

**Figure 1:**
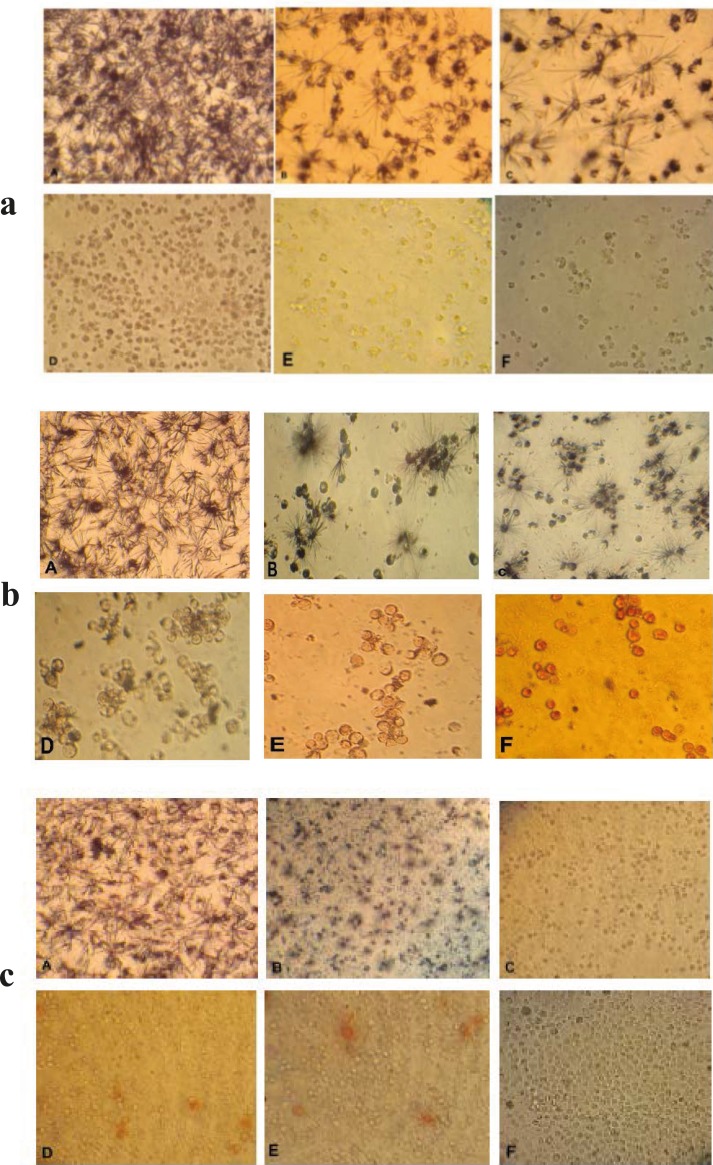
Morphological effect control groups and groups treated with concentrations of 15, 31, 62, 125, 250 µg/ml brittle star dichloromethane extract *O. erinaceus* (**a**) and doxorubicin (**b**) 24 h after treatment observed by inverted microscope (magnification × 200) A: control, B: 15 µg/ml, C: 31 µg/ml, D: 62 µg/ml, E: 125 µg/ml, F: 250 µg/ml. **c**) Morphological changes under treatment with co-administration of brittle star dichloromethane extracts and doxorubicin after 24 h treatment. A: control group, B: 7.5 µg/ml DCM and 7.5 µg/ml doxorubicin (IC_50_) C: 31 µg/ml DCM and 7.5 µg/ml doxorubicin D: 7.5 µg/ml DCM and 31 µg/ml doxorubicin E: 15 µg/ml DCM and 31 µg/ml doxorubicin F: 31 µg/ml DCM and 15 µg/ml doxorubicin. (Magnification × 200).

**Figure 2:**
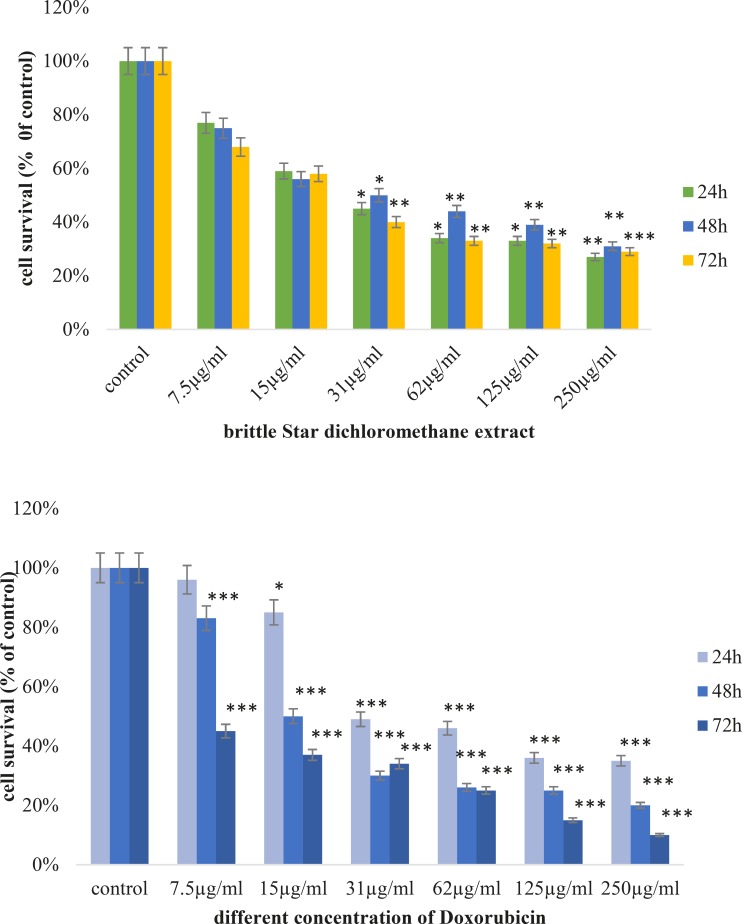
Anti-proliferative activity of brittle star dichloromethane extract and Doxorubicin on EL4 cell line after 24, 48 and 72h treatment, as compared to control. MTT assay (*P<0.05, **P<0.02 and ***P<0.001).

**Figure 3:**
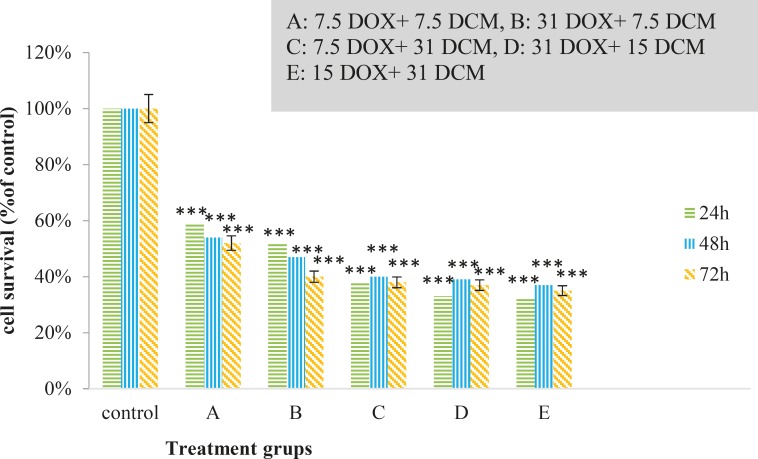
Anti-proliferative activity of synergism treatment by brittle star dichloromethane and Doxorubicin on EL4 cell line after 24, 48 and 72h treatment, as compared to control. MTT assay (*P<0.05, **P<0.02 and ***P<0.001).

Cytotoxic effects of brittle star dichloromethane extract and doxorubicin were evaluated using MTT assay. Inverted microscopy method was used to morphological study of EL4 cell line after treatment with different concentrations of brittle star dichloromethane extract (Figure 1a), Doxorubicin (Figure 1b) and the combination of both of them (Figure 1c). EL4 cells were categorized into two groups; the control group (culture medium RPMI 12%) and treated group. The results showed that control cells are alive, because these formazan crystals formed in all cells, but with increasing concentrations of brittle star dichloromethane extract *O. erinaceus*, the number of crystals reduced so treatment with 31 µg/mL decreased the crystal formation approximately as 50%. At the concentrations of 62, 125, 250 µg/mL none crystals showed cell lysis. The results of MTT exhibited suppression of cell growth under treatment with brittle star extract and doxorubicin in a dose and time depended manner. Therefore, IC_50_ concentrations of brittle star dichloromethane extract (*P˂0*.05), Doxorubicin (*P*˂0.001), and the combination of both of them were observed in 31 and 7.5 µg/mL, respectively (Figure 2, 3). 

*Annexin V-FITC*

**Figure 4.**
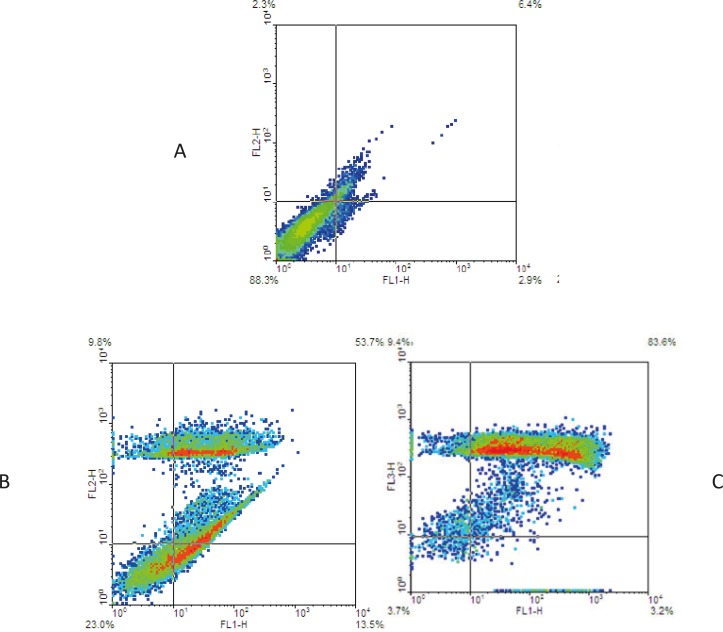
Apoptosis induced by brittle star dichloromethane fraction on EL4 cells. (A) Control (B) 31 and (C) 62 µg/ml of extract conducted by Annexin V/PI assay

To detection induced cell mortality, EL4 cells were treated with 31 and 62 µg/mL of brittle star dichloromethane extract for 24 h. Cells were stained with Annexin V-FITC, according to the manufacturer’s manual, and then were analyzed using flow cytometry. Previous studies reported doxorubicin can induce apoptosis cell mortality in EL4 leukemia cells. The results showed 53.7% of EL4 cell treatment with the concentration of 31 µg/mL brittle star dichloromethane undergoing apoptosis cell mortality. The data showed that brittle star dichloromethane extract induced apoptosis in EL4 cells (Figure 4).

*PI assay*

**Figure 5:**
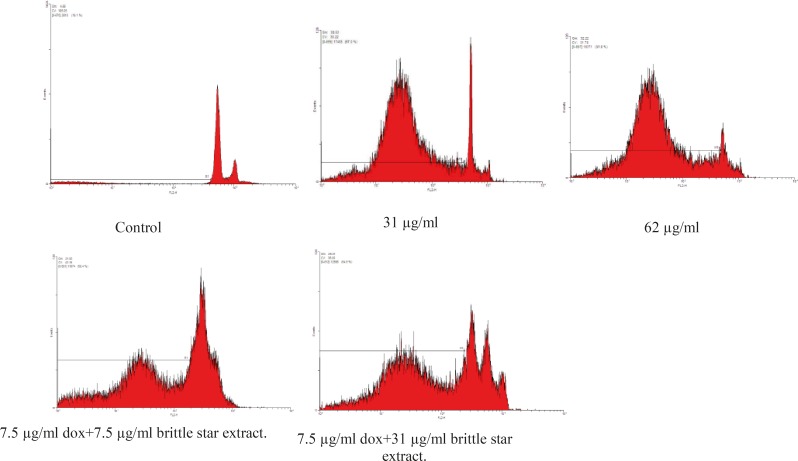
Flow cytometry assessed apoptosis induced by brittle star dichloromethane extract and synergic treatment brittle star dichloromethane extract and Doxorubicin on cells EL4 after staining with PI

Results showed brittle star dichloromethane extract species that can induce apoptosis on EL4 cell line (their sub-G1 peak increased in apoptotic cell). Further, the combination treatment with different concentrations of Doxorubicin and brittle star dichloromethane extract showed more sub-G1 peak compared to the brittle star dichloromethane extract (Figure-5).

*Acridine orange/ propodium iodide staining*

**Figure 6. a,b.c:**
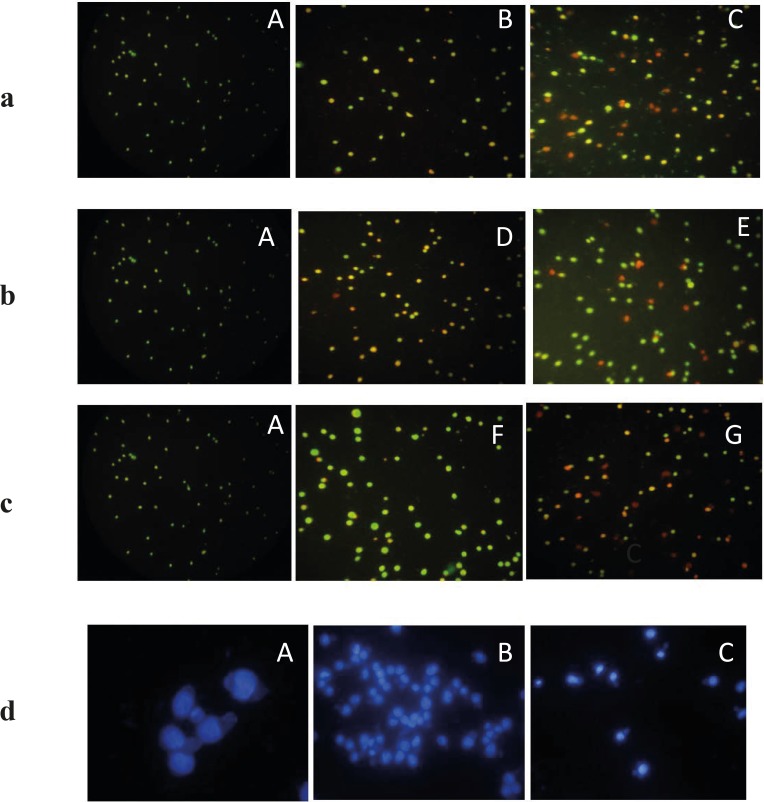
The fluorescence images shows AO/PI staining for induction of apoptosis on EL4 cells whit brittle star dichloromethane extract, Doxorubicin and synergic treatment different concentration Doxorubicin and brittle star dichloromethane extract (31, 62 and 7.5 DOX+ 7.5 DCM and 7.5 DOX+31 µg/mL DCM). (**A)**Control cells and (**B**) 31 µg/mL DCM (**C**) 62 µg/mL DCM (**D**), 31 µg/mL DOX and (**E**) 62 µg/mL DOX, (**F**) 7.5 DOX+7.5 DCM , (G) 7.5 DOX+31 DCM µg/mL (Magnification ×200).**d**) Fluorescence microscopic image of DAPI staining. A (control) B and C (treated EL4 cells with different concentration brittle star dichloromethane extract). (Magnification ×200).

The morphological changes were evaluated by fluorescence microscopy. EL4 cells were stained with alcidine orange/PI after treatment. Red and green colors in nucleus indicate dead and alive cells, respectively (Figure 6). Treated cells displayed late apoptosis (necrotic death) in higher concentration (62 µg/mL) and early apoptosis in less concentrations of extract (31 µg/mL).

*DAPI staining*

DPAI staining showed that EL4 cells treated by different concentrations of brittle star dichloromethane extract, showed non-uniform plasma membrane and DNA fragmentation compared to untreated cells with intact nucleus (Figure 6). 

*Caspase-3 assay and Caspase-9 colorimetric assay*

**Figure 7:**
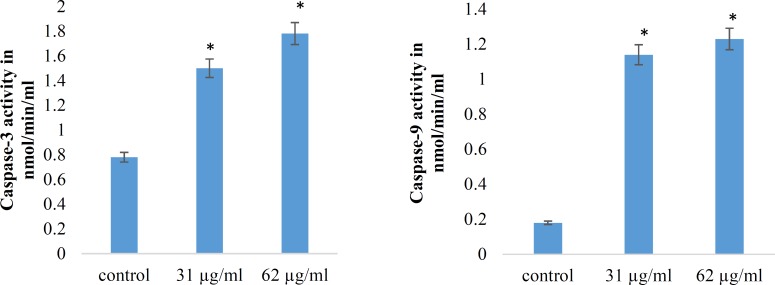
Effect of brittle star dichloromethane extract on Caspase-3 and caspase-9 activities in treated EL4 cells. (A) Histogram represents caspase-3 activity in untreated control and brittle star dichloromethane extract (31, 62 µg/mL). (B) Histogram represents caspase-9 activity in untreated control and brittle star dichloromethane extract (31, 62 µg/mL).

**Figure 8:**
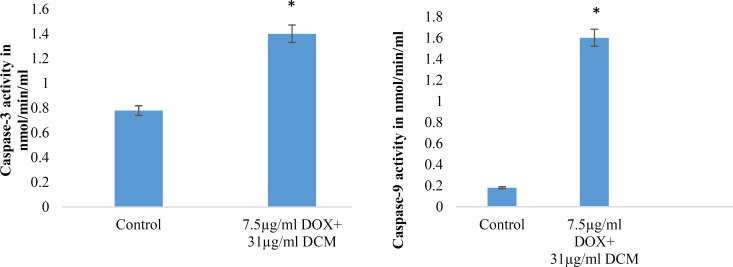
Synergic effect of brittle star dichloromethane extract and Doxorubicin on Caspase-3 and 9 activity in EL4 cells. (**A**) Histogram represents caspase-3 activity in untreated and synergic treated EL4 cells whit brittle star dichloromethane extract and Doxorubicin (31+7.5 µg/mL) treated EL4 cells. (B) Histogram represents caspase-9 activity in untreated control and brittle star dichloromethane extract (31+7.5 µg/mL).

Caspases are cysteine proteases classified as apoptosis executioner (Caspase-3, -6,-7) and apoptosis activator (Caspase-8, -9, -10). Caspas-3 and Caspas-9 involved in mitochondrial pathway. Figures-7 and 8 have shown the activity of Caspase-3 and Caspas-9, increased in a dose dependent under treatment with brittle star dichloromethane extract, and simultaneous treatment with doxorubicin. The results showed that brittle star dichloromethane extract alone and in combination with doxorubicin induced apoptosis through intrinsic pathway in EL4 cells.

In the present study, the morphological observation and MTT assay exhibited that the brittle star dichloromethane extract, same Doxorubicin, has an anti-proliferative activity (IC_50_=31 µg/mL) on EL4 cells in a dose-time depended manner. Further, the PI, Annexin V-FITC, DAPI, Acridine Orange/propodium iodide assay indicated that brittle star dichloromethane extract can induce apoptosis. Furthermore, measuring Caspase-3 and Caspase-9 enzymatic activity revealed that apoptosis induction was performed via caspase dependent pathway that verifies the anticancer potential of marine echinoderm. In this context, some reports have been confirmed anti-carcinogenic capacity of marine invertebrates.

Mutee*et al*. (2012) reported apoptosis induction capacity of a sea star’s (*Acanthasterplanci)* extract which can inhabit MCF-7 cell growth in IC_50_=15.6 μg/mL. The results of the study explained that this apoptotic response is stronger and earlier than the apoptotic effect induced by tamoxifen (1).

Further, they evaluated anticancer activity of *Acanthester planci* extract on MCF-7 (human breast cell line) and HCT-116 (colon cancer cell line), so that obtained results from MTT assay showed that PBS extract demonstrated very potent cytotoxic activity, against both MCF-7 and HCT-116 cell lines, with IC_50_ of 13.48 μg/mL and 28.78 μg/mL, respectively; compared to chloroform extract(with IC_50_ = 121.37 μg/mL (MCF-7) and 77.65 μg/mL (HCT-116) and methanol extract (with IC_50_ = 46.11 μg/mL (MCF-7) and 59.29 μg/mL (HCT-116) (19).

In another study, Prabhu* et al*. (2013) reported the antimicrobial, hemolytic and cytotoxic properties of crude extract of brittle star *Ophiocnemis marmorata. *They found that the cytotoxic activity of the extract is associated to the steroidal compounds existed in the crude extract (20). Further, Levina *et al*. (2012) found that steroid compounds extracted from pacific Starfish *Mithrodiaclavigera* have inhibitory effect on Human Melanoma cells. As a consequence, some of the extracted compounds showed stronger effect on all cell lines of melanoma, whereas others showed no specific effect on these cell lines (21). Althunibat *et al*., (2013) examined antioxidant and cytotoxic properties of two sea cucumbers, *Holothuria edulis Lesson* and *Stichopus horrens Selenka* against A549 and TE1 cancer cells and found that the organic extract of *S. horrens* has more cytotoxic effects against A549 (IC_50_=15.5 μg/mL) and TE1 (IC_50_=4.0 μg/mL) cancer cells (22) compared to Aqueous extract. In another study*, *Timofey*et al*. (2014) studied anticancer activity of Asterosaponins, extracted from the Eastern starfish *Leptasterias ochotensis*, and demonstrated that some of these compounds can significantly inhibit proliferation of cancer cell lines RPMI-7951 and T-47D (23). In a previous study, it was indicated that Methanolic extract of *Asterina pectinifera* has a bioactive effect *on *RAW264.7 (murine leukemia cell line) cell line (Jo *et al*., 2010). Data of the study showed that the methanol extracts (IC_50_=250µg/mL) can induce more cell cytotoxicity than the aqueous extract (IC_50_=1000 µg/mL) (24). 

The results, according to marine natural products, showed that brittle star dichloromethane extract has cytotoxic effect that can be useful as an anticancer candidate for treating blood cancer. Examining morphometric and fluorometric characters suggested that the extract may be introduced as an apoptotic inducer on EL4 cells in 62 µg/mL. As a result, dichloromethane extract revealed a dose depended antigrowth effect against EL4 cells. Therefore, the brittle star dichloromethane has chemo sensitivity of EL4 leukemia cancer cells under treatment with doxorubicin. 

## References

[1] Mutee AF, Salhimi SM, Ghazali FC, Al-Hassan FM, Lim CP, Ibrahim K, Asmawi MZ (2012). Apoptosis induced in human breast cancer cell line by Acanthaster planci starfish extract compared to tamoxifen. Afr. J. Pharm. Pharmacol.

[2] Ebrahimi M, Allahyari A, Ebrahimi M, Mostafavi-Toroghi H, Hosseini G, Karimi M, Rezaiean A, Kazemi MR (2016). Effects of dietary Honey and Ardeh combination on chemotherapy induced gastrointestinal and infectious complications in patients withacute myeloid leukemia: A double-blind randomized clinical trial. IJPR.

[3] Gocek E, Marcinkowska E (2011). Differentiation therapy of acute myeloid leukemia. Cancers (Basel).

[4] Rathee T, Vashist M, Kumar A, Singh S (2014). Incidence of acute and chronic forms of leukemia in Haryana. Int. J. Pharm. Pharm. Sci.

[5] Pokharel M (2012). Leukemia: A review article. Int. J. Adv. Res. Pharm. Bio. Sci.

[6] Suarez-Jimenez GM, Burgos-hernandez A, Ezquerra-Brauer JM (2012). Bioactive peptides and depsipeptides with anticancer potential: sources from marine animals. Mar. Drugs.

[7] Kovar L, Etrych T, Kabesova M, Subr V, Vetvicka D, Hovorka O, Strohalm J, Sklenar J, Chytil P, Ulbrich K, Rihova B (2010). Doxorubicin attached to HPMA copolymer via amide bond modifies the glycosylation pattern of EL4 cells. Tumor Biol.

[8] Octavia Y, Tocchetti CG, Gabrielson KL, Janssens S, Crijns HJ, Moens AL (2012). Doxorubicin-induced cardiomyopathy: from molecular mechanisms to therapeutic strategies. J. Mol. Cell Cardiol.

[9] Sawadogo WR, Schumacher M, Teiten M, Cerella C, Dicato M, Diederich M (2013). A survey of marine natural compounds and their derivatives with anti-cancer activity reported in 2011. Molecules.

[10] Kaur R, Kapoor K, Kaur H (2011). Plants as a source of anticancer agents. Sch. Res. Libr.

[11] Edwards V, Benkendorff K, Young F (2012). Marine compounds selectively induce apoptosis in female reproductive cancer cells but not in primary-derived human reproductive granulosa cell. Mar. Drugs.

[12] Arizza V (2013). Marine biodiversity as source of new drugs. Ital. J. Zool.

[13] Sankari SL, Masthan KMK, Babu NA, Bhattacharjee T, Elumalai M (2012). Apoptosis in cancer - an update. Asian Pacific J. Cancer Prev.

[14] Kakanj M, Vatanpour H, Ghazi-Khansari M, Zare Mirakabadi A, Daraei B (2015). Cytotoxic Effect of Iranian Vipera Lebetina snake Venom on HUVEC Cells. IJPR.

[15] Demain AL, Vaishnav P (2011). Natural products for cancer chemotherapy. Microb. Biotechnol.

[16] Zoysa M De (2012). Medicinal benefits of marine invertebrates: sources for discovering natural drug candidates. Adv. Food Nutr. Res.

[17] Smith LC, Ghosh J, Buckley KM, Clow LA, Dheilly NM, Haug T, Henson JH, Li C, Lun CM, Majeske AJ, Matranga V, Nair SV, Rast JP, Raftos DA, Roth M, Sacchi S, Schrankel CS, Stensvåg K (2010). Echinoderm immunity. Adv Exp Med Biol.

[18] Suguna A, Bragadeeswaran S, Prabhu K, Priyatharsini S, Mohanraj M, Sivaramakrishnan S (2013). Cytolytic and antinociceptive activities of starfish Protoreaster linckii (Blainvilli, 1893). Afr. J. Pharm. Pharmacol.

[19] Mutee AF, Salhimi SM, Ghazali FC, Aisha AF, Lim CP, Ibrahim K, Asmavi MZ (2012). Evaluation of anti-cancer activity of Acanthester planci extracts obtained by different methods of extraction. Pak. J. Pharm. Sci.

[20] Prabhu K, Bragadeeswaran S (2013). Biological properties of brittle star Ophiocnemis marmorata collected from Parangipettai, Southeast coast of India. J. Microbiol. Antimicrob.

[21] Levina E V, Kalinovskii AI, Ermakova SP, Dmitrenok PS (2012). Steroid compounds from pacific starfish Mithrodia clavigera and their toxicity to human melanoma cells. Russ. J. Bioorganic Chem.

[22] Althunibat YO, Ridzwan BH, Taher M, Daud J, Ichwan SUA, Qarlleh H (2013). Antioxidant and cytotoxic properties of two sea cucumbers, Holothuria edulis Lesson and Stichopus horrens Selenka. Acta Biol. Hung.

[23] Malyarenko TV, Kicha AA, Ivanchina NV, Kalinovsky AI, Popov RS, Vishchuk OS, Stonik VA (2014). Asterosaponins from the ar eastern starfish Leptasterias ochotensis and their anticancer activity. Steroids.

[24] Jo WS, Choi YJ, Kim HJ, Nam BH, Lee GA, Seo SY, Lee SW, Jeong MH (2010). Methanolic extract of Asterina pectinifera inhibits LPS-induced inflammatory mediators in murine macrophage. Toxicol. Res.

